# Editorial: Design of Macrocyclic Compounds for Biomedical Applications

**DOI:** 10.3389/fchem.2021.730111

**Published:** 2021-07-08

**Authors:** Pavel L. Padnya, Xin Wu, Andrea Erxleben, Susana S. Braga

**Affiliations:** ^1^ A.M. Butlerov' Chemistry Institute, Kazan Federal University, Kazan, Russia; ^2^ School of Chemistry, The University of Sydney, Sydney, NSW, Australia; ^3^ School of Chemistry, National University of Ireland Galway, Galway, Ireland; ^4^ LAQV/REQUIMTE – Associated Laboratory for Green Chemistry, Department of Chemistry, University of Aveiro, Aveiro, Portugal

**Keywords:** macrocycles, self-assembly, molecular recognition, drug delivery, biosensors

The search for new biologically active compounds to tackle global health challenges is an important goal in organic synthesis, biochemistry, and medicine. The biological activity of macrocyclic structures is an actively developing area of science. Macrocyclic compounds have unique physicochemical properties, such as spatial preorganization, presence of a size-defined cavity, propensity of undergoing self-assembly, low pharmacologically active concentrations, low toxicity, antiallergenicity, etc. Macrocyclic compounds such as crown ethers, cyclodextrins, cucurbiturils, calixarenes, porphyrins, pillararenes and cyclic peptides have been used in various biomedical applications. In this Research Topic, we present a collection of original research and review articles that show the significant recent advances made in the synthesis of new macrocycles and their biomedical applications.

One of the ways to overcome the antimicrobial resistance of microorganisms is the use of macrocycle-antibiotic hybrids, as reviewed by Surur et al.. This review article describes the synthesis and properties of the most well-known hybrids of macrocyclic compounds with antibiotics such as rifamycins, vancomycin, etc. It is known that the major limitation of the use of antibiotic hybrids is the increase in molecular weight. However, progresses and advances in this area show the possibility of improving the oral bioavailability of bulky molecules for systemic clinical use.

Ibrahim et al. have developed novel Tröger’s base derivatives and studied their anticancer properties. The synthesized Tröger’s base phenhomazines derivatives were screened for anticancer activity, and 1,4,7,10-tetraoxa[10](2,8) trögerophane has shown a promising selectivity on a colon cell line with IC_50_ = 92.7 μg/ml. The obtained results open up prospects for the development of new promising anticancer agents.

It is known that macrocyclic compounds can be used in targeted delivery systems as containers for drugs. It has been shown by Chandra et al. that cucurbit[7]uril is able to form a host-guest complex with the neurotransmitter serotonin. In this case, the binding affinity is pH-dependent. The results demonstrate promising biological applications in the delivery and pH-controlled release of serotonin. Saleh et al. described the use of cucurbit[7]uril as a liver protective agent and an adjuvant in toxicological pharmacology. The protective effects of cucurbit[7]uril were evaluated in the biochemical study of the extracted livers of mice following cyanobacterial crude extract treatment with or without cucurbit[7]uril. The addition of cucurbit[7]uril has been shown to significantly reduce the toxicity of cyanotoxin-induced hepatotoxicity (*P* < 0.05) *in vivo*.

Supramolecular chemistry is useful in the controlled and/or local delivery of immunomodulatory drugs, as reviewed by Soni et al.. In their review, macrocycles such as cyclodextrins are highlighted for their drug solubilizing and stabilizing actions and the utility of polymer-based hydrogels and nanomaterials in local drug delivery is also described. Besides drug delivery, cyclodextrin-based supramolecular systems have found applications in other biomedically relevant areas such as the separation of biomolecules, enzymatic catalysis, sensing, diagnosis and therapy, as summarized by Luo et al.

The chirality of cyclodextrins makes them suitable for the separation of biomolecules. According to the molecular mechanics simulation conducted by Alvira, even a very simple and affordable native cyclodextrin such as β-cyclodextrin is able to discriminate the enantiomeric forms of the amino acids alanine, valine, leucine, and isoleucine.

The development of macrocyclic chemistry has contributed to the design of new materials with effective and selective catalytic properties. In a mini-review, Shang et al. have described recent advances in the use of macrocyclic compounds as building blocks for the design of bioinspired catalysts. The described materials, from single-molecule to metal-organic framework materials, have unique catalytic properties and binding affinity for biologically significant substrates. The authors agree that the materials obtained have a lower catalytic activity in comparison with natural enzymes at this stage of research. However, the trend in the development of synthetic catalysts may lead to future application.

Facilitation of ion transport across lipid bilayers is an important supramolecular function with potential applications in biophysics research and the treatment of ion channel diseases. Macrocyclic ionophores are of particular interest for achieving high membrane transport selectivity as exemplified by the almost perfect K^+^ over Na^+^ selectivity exhibited by the natural product valinomycin. Zhao et al. have developed a cyclic azapeptide anionophore with a small binding cavity that facilitates selective transmembrane transport of fluoride ions. Remarkably, this anionophore shows negligible transport for larger anions including chloride and acetate.

Overall, this article collection includes several excellent studies in the design and investigation of macrocyclic structures and covers various fields of chemistry, biology and medicine. As guest editors of this article collection, we thank all authors and reviewers for their valuable contributions. We hope that the publications presented in this collection will attract even greater interest in the chemistry of macrocyclic compounds and supramolecular chemistry.

